# Usefulness of Choline-PET for the detection of residual hemangiopericytoma in the skull base: comparison with FDG-PET

**DOI:** 10.1186/1746-160X-8-3

**Published:** 2012-02-07

**Authors:** Shin Ito, Junkichi Yokoyama, Hitoshi Yoshimoto, Masaki Yazawa, Kubota Kazuo, Makoto Hanaguri, Shinichi Ohba, Mitsuhisa Fujimaki, Katsuhisa Ikeda

**Affiliations:** 1 Department of Otolaryngology, Head and Neck Surgery, Juntendo University School of Medicine, Tokyo, Japan; 2 Department of Oral maxillofacial surgery, Osaka Dental University School of Dentistry, Osaka, Japan; 3 Department of Plastic Surgery, Keio University School of Medicine, Tokyo, Japan; 4 Division of Nuclear Medicine, Department of Radiology, International Medical Center of Japan, Tokyo, Japan; 5 Department of Otolaryngology, Kyushu Rosai Hospital, Kitakyushu, Japan; 6 2-1-1 Hongo, Bunkyo-ku, Tokyo 113-8421, Japan

**Keywords:** Choline-PET, Skull base tumor, Hemangiopericytoma, Skullbase surgery

## Abstract

**Background:**

Choline is a new PET tracer that is useful for the detection of malignant tumor. Choline is a precursor of the biosynthesis of phosphatidylcholine, a major phospholipid in the cell membrane of eukaryotic cells. Malignant tumors have an elevated level of phosphatidylcholine in cell membrane. Thus, choline is a marker of tumor malignancy.

**Method:**

The patient was a 51-year-old man with repeated recurrent hemangiopericytoma in the skull base. We performed Choline-PET in this patient after various treatments and compared findings with those of FDG-PET.

**Results:**

Choline accumulated in this tumor, but FDG did not accumulate. We diagnosed this tumor as residual hemangiopericytoma and performed the resection of the residual tumor. FDG-PET is not appropriate for skull base tumor detection because uptake in the brain is very strong.

**Conclusion:**

We emphasize the usefulness of Choline-PET for the detection of residual hemangiopericytoma in the skull base after various treatments, compared with FDG-PET.

## Background

Positron emission tomography (PET) has been successfully used for the diagnosis of head and neck cancer and 18-fluorodeoxyglucose (FDG) is the most commonly used PET tracer and FDG-PET offers the most effectiveness for imaging various tumors. However, this technique is not appropriate for skull base tumor detection because FDG uptake in the brain is very strong. Recently 11 C-choline was developed as a new PET tracer. Here we report the effectiveness of this tracer for PET imaging of residual hemangiopericytoma in the skull base. Hemangiopericytoma is a very rare tumor in the head and neck region. This tumor sometimes relapse in the base of skull. Repeated surgeries could cause severe postoperative complications. Therefore, accurate diagnosis of recurrent tumor is strongly required now.

## Case report

**Figure 1:**
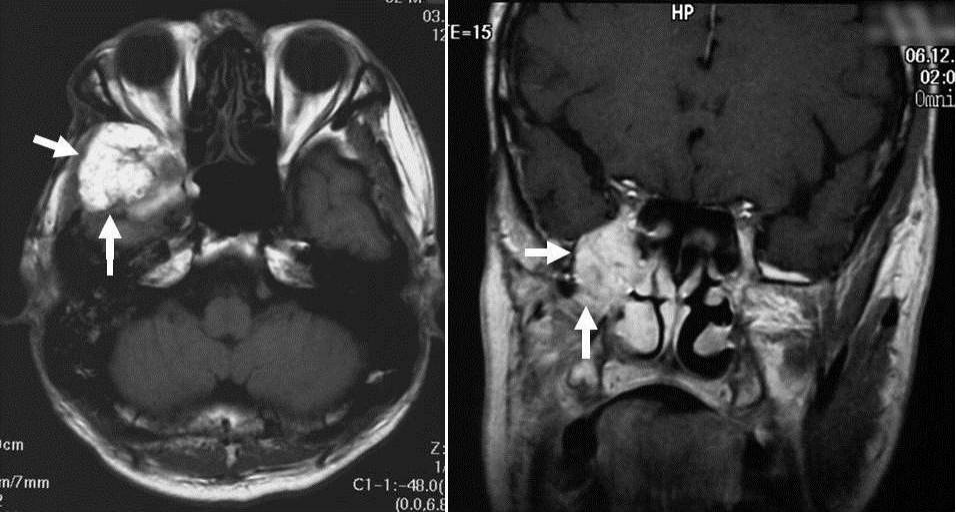
**MRI findings (axial section and coronal section): The tumor (arrow) in the right temple showed low signal intensity on T1 weighted image (left) and enhanced by the contrast medium**. (right).

**Figure 2:**
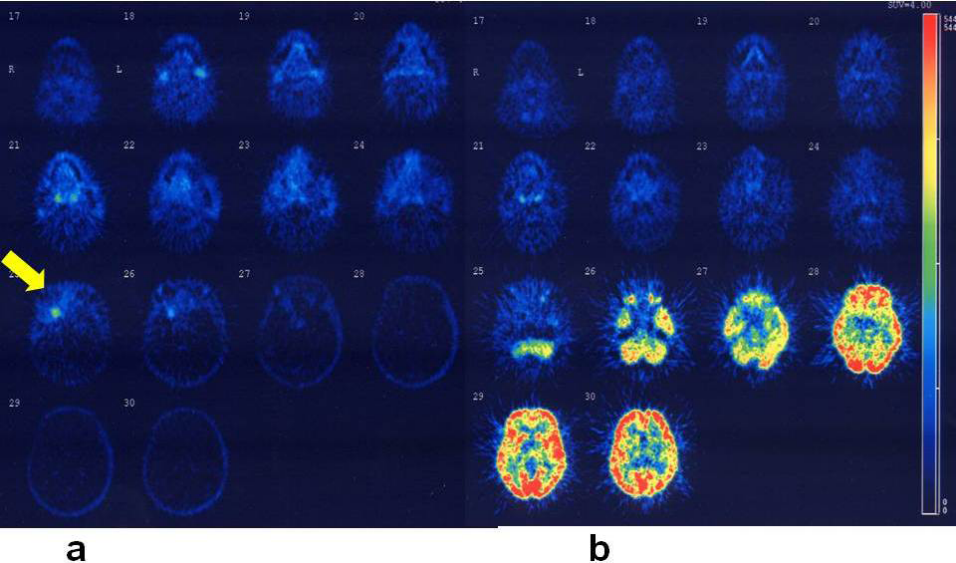
**Result of PET study (left side:Choline-PET, right side: FDG-PET)**. Choline-PET (left side) showed the recurrent tumor in the tip of the right temporal lobe (arrow) but FDG-PET (right side)could not detect the tumor. SUV max 4.

**Figure 3:**
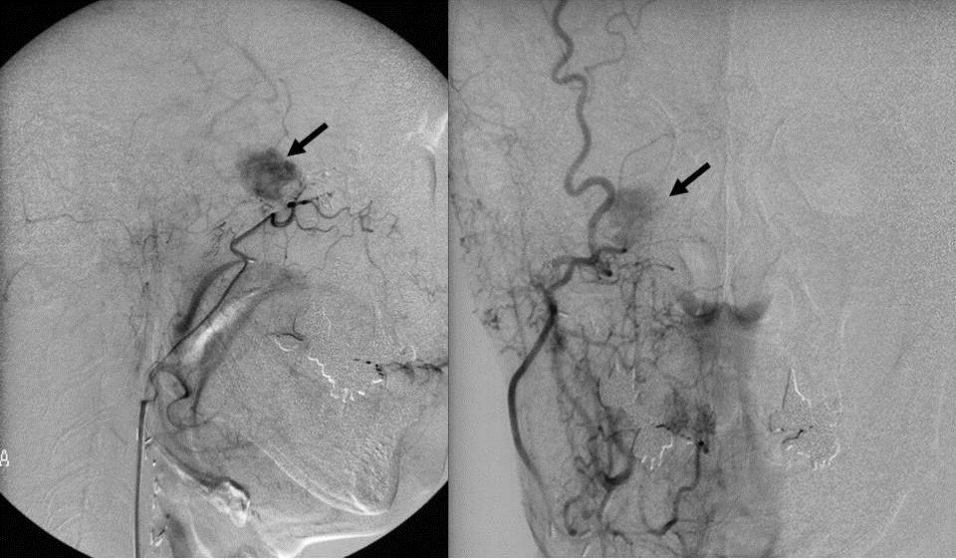
**Angiography findings: Lateral and Frontal view of right external carotid artery angiography showed demonstrated the lesion strongly**.

**Figure 4:**
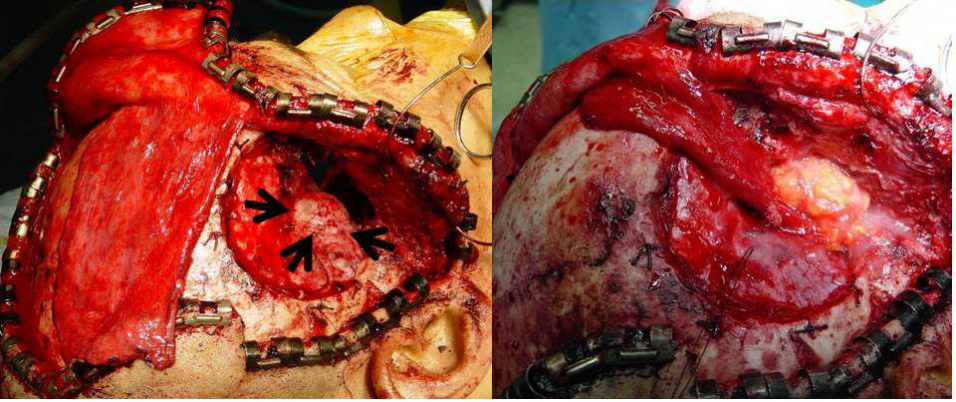
**Operative findings**. The tumor was completely resected together with a part of dura from the right temple (right). The skull base was reconstructed by galeal flap and the dead space was plugged with abdominal fat tissue (left).

**Figure 5:**
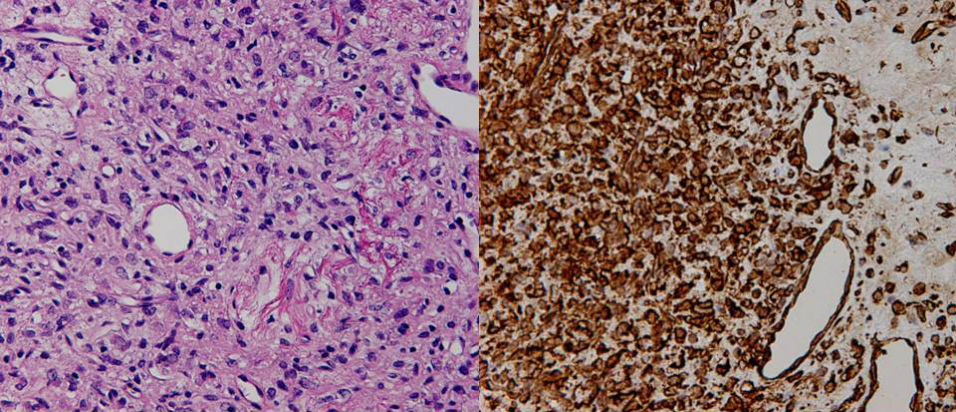
**Pathologic finding: a: This tumor was composed of round or spindle-shaped cells, including dilated staghorn-shaped vessels (H-E × 200)**. b: Immunohistochemical staining of Vimentin showed positive expression in cancer cells. c: Expressions of CD34(+) in cancer cells were positive on immunostaning.

A 51-years-old Japanese man presented with a tumor in the right temple and underwent resection of the tumor at a medical college hospital in 1979. The histological diagnosis was hemangiopericytoma. Thereafter, he demonstrated local recurrence many times, underwent resection 5 times and embolization was performed 2 times. Despite these treatments, this tumor relapsed in the temple again and was treated by irradiation (total 61.8 Gy) in November 2002. However, the tumor appeared to persist and multiple lung metastases were detected. After 4 months, he consulted our hospital for their treatment. On the first medical examination at our hospital, we evaluated the skull base tumor with MRI (Figure 1) and lung metastasis with chest-CT. Because lung metastasis remained stable, we performed Choline-PET and compared findings to those of FDG-PET. Choline accumulated in the tip of the right temporal lobe (SUV max 4.0) but FDG-PET could not detect in the tumor because it accumulated strongly throughout the whole brain without demonstrating the tumor (Figure 2). In addition to PET study, angiography via the right external carotid artery demonstrated the lesion strongly (Figure 3). Based on these examinations, we diagnosed this lesion as residual hemangiopericytoma. Then he underwent resection of the residual skull base tumor in February 2004. First, we incised the coronary line at the front of the head, and tried to approach this tumor via right temporal craniotomy without facial incision. We resected completely the tumor together with a part of dura because of dural invasion. The tumor showed diffuse invasion of tissue around the mass lesion. We reconstructed the skull base by galeal flap. In addition, we plugged the dead space after tumor resection with abdominal fat tissue (Figure 4). There were no complications postoperatively. This tumor demonstrated abundant blood vessels and was composed of round or spindle-shaped cells, including dilated staghorn-shaped vessels (Figure 5). Immunohistochemically, expressions of Vimentin and CD34(+) in cancer cells were positive on immunostaning, while these of S-100 protein and cytokeratin were negative(Figure 5). This histological diagnosis was recurrent hemangiopericytoma. The patient demonstrated a favorable postoperative course and was discharged from our hospital on March 2004. To date, we have not detected any recurrence in the skullbase for 7 years postoperatively but lung metastases increased gradually. Currently, he can work as well as he could before onset of the disease.

## Discussion

FDG-PET is not appropriate for skull base tumor detection or evaluation of recurrence because FDG uptake throughout the brain is very strong. We used Choline-PET, which was developed by Hara et all [1]. Choline is a precursor of the biosynthesis of phosphatidylcholine, which is a major phospholipid in the cell membrane of eukaryotic cell. Malignant tumors have an elevated level of phosphatidylcholine in their cell membranes. Thus, choline is a marker of malignant tumor. Choline has also a tendency to accumulate significantly in the liver, kidney and spleen as well as the pancreas. Therefore Choline-PET would not be useful in the upper abdomen. The features of Choline-PET include a shorter examination period and lower uptake in the muscle and brain than those PET with other tracers. Choline-PET has been reported to be highly effective in imaging lung cancer [2], brain tumors [3], prostate cancer [4] and esophageal cancer [5]. There is a similarity between skull base tumors and brain tumors. Thus, we performed Choline-PET to malignant skull base tumor in this case, although there were no reports describing Choline-PET for head and neck tumor. In the future, we will further investigate the usefulness of Choline-PET in head and neck tumor patients in comparison with that of FDG-PET in our hospital. Herein, present the first report of recurrence of hemangiopericytoma demonstrated by Choline-PET but not FDG-PET.

Hemangiopericytoma is a rare tumor of the pericytes of Zimmermann [6], which was first described by Stout and Murray [7] in 1942. On histopathologic examination, the tumor is characterized by a proliferation of oval and spindle-shaped pericytic cells. The largest series was published by Enzinger and Smith [8] and analyzed 106 cases of hemangiopericytoma and 16% of these patients had lesions in the head and neck region. The first choice of treatment for hemangiopericytoma is wide surgical excision. Surgical excision is the preferred treatment, but recurrence and metastasis can subsequently occur in up to 50% of cases [9]. On first diagnosis, it is difficult to distinguish between benign and malignant forms. Recurrence may develop after a prolonged disease-free interval, which stresses the need for long-term follow-up. Therefore, we should detect recurrence as early as possible, then resect the recurrence completely without causing postoperative dysfunction.

We were able to diagnose residual hemangiopericytoma with Choline-PET after radiation therapy and could completely resect this tumor safely. Although the lung metastases have slightly increased after the treatment, the patient maintains a high quality of life now. This was a repeatedly recurrent case, therefore the patient should be carefully followed.

## Conclusion

We emphasize the usefulness of Choline-PET for the detection of residual hemangiopericytoma in the skull base after various treatments, compared with FDG-PET.

## Consent statement

Written informed consent was obtained from the patient for publication of this case report and accompanying images. A copy of the written consent is available for review by the Editor-in-Chief of this journal.

## Funding

Supported in part by a Grant for Clinical Cancer Research from the Ministry of Health, Labor, and Welfare of Japan.

## Competing interests

The authors declare that they have no competing interests.

## Authors' contributions

JY and SI contributed equally to this work. HY, MY and SO conceived of the study and participated in its design and coordination. KK and MF drafted the manuscript. JY and KI were involved in revising the manuscript. All authors read and approved the final manuscript.
